# PA6 Stromal Cell Co-Culture Enhances SH-SY5Y and VSC4.1 Neuroblastoma Differentiation to Mature Phenotypes

**DOI:** 10.1371/journal.pone.0159051

**Published:** 2016-07-08

**Authors:** Ross Ferguson, Vasanta Subramanian

**Affiliations:** Department of Biology and Biochemistry, University of Bath, Bath, United Kingdom; INSERM U894, FRANCE

## Abstract

Neuroblastoma cell lines such as SH-SY5Y have been used for modelling neurodegenerative diseases and for studying basic mechanisms in neuroscience. Since neuroblastoma cells proliferate and generally do not express markers of mature or functional neurons, we exploited a co-culture system with the stromal cell line PA6 to better induce differentiation to a more physiologically relevant status. We found that co-culture of the neuroblastoma cell lines in the presence of neural inducers such retinoic acid was able to generate a high proportion of quiescent neurons with very long neurites expressing differentiation markers. The co-culture system additionally cuts short the time taken to produce a more mature phenotype. We also show the application of this system to study proteins implicated in motor neuron disease.

## Introduction

Investigation into fundamental mechanisms of neuroscience or therapeutic interventions can be rapidly and reproducibly performed using *in vitro* models such as immortalised, easily handled established neuroblastoma cell lines. With this comes the problem of cell line identity as neuroblastoma cells which are typically not quiescent, lack extensive neurite projections and key marker expression characteristic of mature neurons. In their default proliferative state neuroblastoma cell lines do not behave experimentally as equivalents to their primary counterparts in response to disease relevant agents such as oxidative stressors or neurotoxins [1–3]. However, if culture conditions can be established to differentiate the neuroblastoma lines to cells with characteristics closer to those of mature neurons these would provide excellent model systems to study neurodegenerative conditions such as motor neuron disease

SH-SY5Y, a subclone of the human neuroblastoma cell line SK-N-SH derived from a metastatic bone tumour, typically shows a proliferative neuroblast-like phenotype [4] and is used extensively as a model for neurodegeneration studies. Since it expresses moderate levels of dopaminergic markers such as tyrosine hydroxylase and dopamine-β hydroxylase [5] it has been a popular dopaminergic model for many years, however their dopaminergic identity and suitability have been questioned [6,7]. VSC4.1 is motor neuron hybrid cell line created through the fusion of a rat ventral spinal cord neuron with a mouse neuroblastoma cell [8]. VSC4.1 has been shown to express the neuronal markers neurofilament-H, synaptophysin and neuron-specific enolase as well as the cholinergic marker choline acetyl transferase [9]. Their motor neuron-like identity has provided a model for motor-neuron diseases (MND) such as amyotrophic lateral sclerosis. However, they are proliferative and have short neurite extensions.

Multiple protocols for differentiation to more mature phenotypes have been developed to circumvent some of these drawbacks. SH-SY5Y is most commonly differentiated by culture in the presence of super-physiological concentrations of retinoic acid (RA) [10] resulting in cholinergic identity with increased choline acetyl transferase activity [11] and vesicular monoamine transporter levels but little change in dopaminergic markers [12]. Conversely, differentiation with factors such as the phorbol ester 12-*O*-tetradecanoyl-phorbol-13-acetate (TPA) alone or in combination with RA have been shown to increase dopaminergic markers such as tyrosine hydroxylase (TH) and dopamine transporter (DAT) therefore increasing sensitivity to 1-methyl-4-phenylpyridinium (MPP+) commonly used to induce Parkinsonism symptoms [12–14][13]. After differentiations under these conditions SH-SY5Y meet many of the criteria of mature neurons, extended culture in the presence of BDNF has been reported to cause exit from the cell cycle [15]. Far fewer protocols exist for the differentiation of VSC4.1. These typically involve extended culture in medium containing dibutyryl cyclicAMP (dbcAMP) [9,16]. Differentiation has been reported to increase expression of neuronal voltage gated calcium channels, choline acetyl transferase, neurofilament-H and synaptophysin. Protocols for both cell lines require from eight to fourteen days before cells are sufficiently mature for use experimentally.

Pre-adipose PA6 cells clonally derived from new-born mouse calvaria [17] have been shown to promote the differentiation of both mouse [18] and human embryonic stem (ES) cells [19] to dopaminergic neurons in co-culture. This property of PA6 has been attributed to factors collectively referred to as stromal cell-derived inducing activity (SDIA) produced by these cells and occurs in the absence of externally added factors such as RA or internal signals generated within embryoid bodies [18]. These factors have been identified to be Wnt5a, stromal cell-derived factor 1 (SDF1), insulin-like growth factor 2 (IGF2), pleiotrophin(PTN), ephrin B1 (EFNB1), sonic hedgehog homolog (Shh), secreted frizzled-related protein 1 (sFRP1) and vascular endothelial growth factor D. Co-culture of ES cells with PA6 also drastically cuts the culture time needed to obtain mid-brain dopaminergic neurons from around 14–20 days [20,21] to between six and eight days.

We have previously exploited PA6 co-culture in conjunction with RA in order to obtain motor neurons from P19 mouse terato-carcinoma cells for use as a model of motor neuron disease (MND) [22,23]. Since PA6 cells produce Shh (which promotes a ventral neuronal identity ie motor neurons) we used the PA6 co-culture system in conjunction with higher concentration of RA (which leads to a posterior neuronal identity) to rapidly differentiate the two neuronal-like cell lines VSC4.1 and SH-SY5Y to a more mature and therefore physiologically relevant motor neurons particularly with a view to using them for studying genes involved in motor neuron disease such as Angiogenin [23,24].

## Methods

### Cell lines and culture

PA6 (provided by Professor Mel Greaves, ICR, London, UK) cells were cultured in alpha-MEM containing deoxynucleosides, Glutamax (Life) 10% Foetal bovine serum (FBS) (Biosera) and 1% Non-essential amino acids (NEAA) (Life) (PA6 complete medium). VSC4.1 (obtained from Professor Angela Vincent, IMM, Oxford) were maintained on culture dishes coated with poly-l-ornithine (PLO) (Sigma) in DMEM:F12 (1:1) containing Glutamax (Life) supplemented with 2% FBS, 1% N1 (Sigma) and 1% NEAA (referred to as VSC4.1 complete medium). SH-SY5Y were obtained from ECACC (Porton Down, UK) and maintained in DMEM:F12 (1:1) containing Glutamax supplemented with 15% FBS and 1% NEAA (SH-SY5Y complete medium). SH-SY5Y expressing HA-tagged mouse angiogenin 1 were generated as previously described [23]

### Differentiation of SH-SY5Y and VSC4.1 without PA6

Retinoic acid (RA) was used to induce the differentiation of SH-SY5Y and dibutyryl cyclic AMP (dbcAMP) for VSC4.1 SH-SY5Y cells were plated on coverslips coated with 0.1% gelatin in 24 well plates in DMEM:F12 (1:1) containing Glutamax supplemented with 1% FBS, 1% NEAA and 10μM RA at a final of 5000 cells each. Media was changed every 48h. VSC4.1 cells were plated on coverslips coated with PLO in 0.5 ml complete medium supplemented with 1mM dbcAMP to a final cell density of 5000 cells per well. Media was changed every 48h.

### Differentiation of SH-SY5Y and VSC4.1 cells in co-culture with PA6

Two days prior to co-culture, subconfluent cultures of PA6 cells are trypsinised, re-suspended in PA6 complete medium and seeded on gelatinised coverslips in 24 well plates (0.5ml/well) at a ratio of ~1:2 (i.e. one T75 flask to three 24 well plates). PA6 cells at the time of plating SH-SY5Y or VSC4.1 should be a 100% confluent monolayer, this is usually achieved in two days. To induce differentiation in co-culture, SH-SY5Y cells were plated on PA6 monolayer on coverslips (5000 cells/0.5ml/well) in 24 well plates in alpha-MEM containing deoxynucleosides and Glutamax supplemented with 1% Knock-out serum replacement (KOSR) (Life Technologies), 1% NEAA and 10μM RA. VSC4.1 cells were also plated at similar densities on PA6 monolayer in alpha-MEM containing deoxynucleosides and Glutamax supplemented with 1% Knock-out serum replacement (KOSR) (Life Technologies), 1% NEAA, 0.5μM RA and 1mM dbcAMP. Media was changed every 48h for both co-cultures.

### Immunocytochemistry

Cells cultured on coverslips were fixed in 4% PFA (Sigma) in PBS (pH7.4) for 15min on ice, washed twice with PBS and incubated at room temperature for one hour in blocking solution (0.1% gelatin, 1% FBS, 0.5% Triton X-100 (BDH) in PBS). Fixed cells were incubated overnight at 4°C with primary antibodies diluted in blocking buffer. Cell monolayers were washed (4X10min) in PBS-Tr (0.1% Triton X-100 in PBS) and incubated for two hours with appropriate secondary antibody and DAPI diluted in blocking buffer. Unbound secondary antibody was removed by PBS-Tr washes (4X10 min), followed by two PBS washes before mounting in Mowiol (Sigma). Stained cells were and imaged on a Leica DMRB microscope. Primary antibodies used for immunocytochemistry and their dilutions are as follows: mouse anti-nestin (Rat401, DSHB) 1:50, mouse anti-NF-M (2H3, DSHB) 1:5, mouse anti-Islet (40.2D6, DSHB) 1:5, rabbit anti-peripherin (Millipore) 1:1000, rabbit anti-MAP2 (Millipore), goat anti-CHAT (Millipore) 1:200, rabbit anti-GAD1 (Millipore) 1:500, rabbit anti-Tyrosine Hydroxylase (Epitomics) 1:500, goat anti-mouse Alexa Fluor 488 (Life Technologies) 1:2000, goat anti-mouse Alexa Fluor 568 (Life Technologies) 1:2000, goat anti-rabbit Alexa Fluor 488 (Life Technologies) 1:2000, goat anti-rabbit Alexa Fluor 594 (Life Technologies) 1:2000. DAPI (Sigma) 1:2000.

### Quantification of neurite length and data analysis

Neurite lengths were measured from two independent experiments for each indicated method using ImageJ (Rasband, W.S., ImageJ, U. S. National Institutes of Health, Bethesda, Maryland, USA, http://imagej.nih.gov/ij/, 1997–2014). Data were analysed in SPSS v21 (IBM). Neurite length data were highly skewed, therefore, they were transformed by log to a normal distribution prior to comparison by t-test. Figs were created in Photoshop CS3 (Adobe).

### RT-PCR

Cells were lysed in Trizol (Life Technologies) and total RNA prepared following manufacturer’s instructions. RNA was treated with DNase (Ambion, DNAfree) and reverse transcribed (Fermentas Revert-Aid H minus first-strand cDNA synthesis kit) using oligodT primers. RT-PCR was performed in 30ul reactions on 10ng template using Promega GoTaq system with 1.5mM MgCl_2_ and 0.5mM of each primer. PCR primers (Table 1) were synthesised by Life Technologies. Cycling conditions were as follows: 95°C for 2min, then 35 cycles of 95°C for 30s, 60°C for 30s (or 55°C for ChAT and TH reactions) and 72°C for 30s, followed by 72°C for 1min before a 10°C hold.

**Table 1:** RT-PCR primer sequences.

Gene	Accession	Forward Primer 5’-3’	Reverse Primer 5’-3’	bp
NES	NM_006617.1	CAGCGTTGGAACAGAGGTTGG	TGGCACAGGTGTCTCAAGGGTAG	389
MAP2	NM_002374.3	CCACCTGAGATTAAGGATCA	GGCTTACTTTGCTTCTCTGA	482
GAD1	NM_000817.2	GTCGAGGACTCTGGACAGTA	GGAAGCAGATCTCTAGCAAA	357
SLC6A4	NM_001045.5	GCCTTTTACATTGCTTCCTA	CCAATTGGGTTTCAAGTAGA	447
ChAT	NM_020985.3	ACTGGGTGTCTGAGTACTGG	TTGGAAGCCATTTTGACTAT	451
TH	NM_199292.2	TCATCACCTGGTCACCAAGTT	GGTCGCCGTGCCTGTACT	125
ACTB	NM_001101.3	ACCATGGATGATGATATCGC	TCATTGTAGAAGGTGTGGTG	281

### Calcium imaging

Cells were differentiated for eight days with our without PA6 co-culture, or plated 48 prior to analysis on black-sided 96well plates (Costar). Cells were incubated for 45 minutes at room temperature in 2μM Fura-2 AM (ThermoFisher) in media (DMEM without phenol red supplemented with 0.1% KOSR (Life Technologies)). Cells were washed once and incubated again with media for 30 minutes at room temperature. Wells were analysed at 1s intervals over 40s with KCl addition at 10s to a final concentration of 50mM. 2μM Ionomycin (ThermoFisher) addition was used as a positive control. Fluorescence was recorded at 510nm after excitation at 340nm and 380nm at 37°C and 5% CO_2_ in a BMG Clariostar plate reader. Ratios were calculated from the means of triplicate wells.

## Results

**Fig 1:**
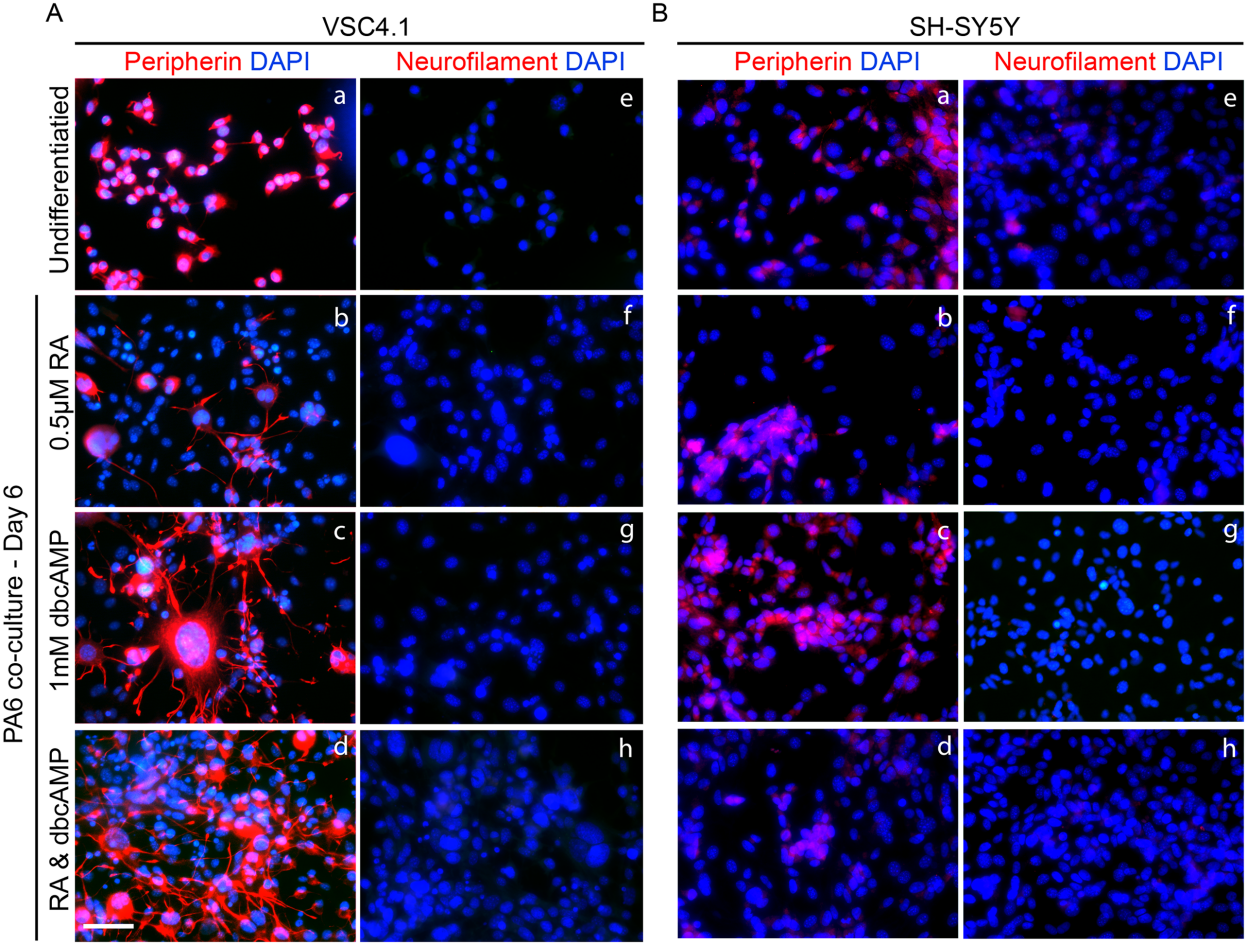
Optimisation of conditions for differentiation of SH-SY5Y and VSC4.1 on PA6. (A) Undifferentiated VSC4.1 have very short peripherin positive processes (a) but not neurofilament (e). Differentiation on PA6 results in extension of peripherin positive neurites on day six (b,d). RA and dbcAMP combined with PA6(c,g) promote extensive neurite outgrowth when compared to either alone (b,f or c,g). No neurofilament staining is seen under any condition. (B) Undifferentiated SY5Y do not express peripherin or neurofilament while undifferentiated or after differentiation on PA6 with 0.5μM RA, 1mM dbcAMP or both combined (a-d). Scale bar 50μm.

We initially optimised conditions for differentiation of VSC4.1 and SH-SY5Y on PA6 and monitored this by staining for some key markers on day 6 of differentiation. Peripherin is a marker for peripheral and motor neurons. It is ubiquitously expressed by VSC4.1 cells. This was used to observe cell morphology and qualify neurite extension during differentiation. Undifferentiated VSC4.1 cells possess peripherin positive cell bodies with short neurites which are no longer than a single cell diameter and uniformly sized nuclei (Fig 1A-a). After six days of differentiation on PA6 in the presence of 0.5μM RA, VSC4.1 cells show slightly enlarged cell bodies relative to undifferentiated controls and neurites extended to between two and three times their undifferentiated length (Fig 1A-b). Differentiation with 1mM dbcAMP alone resulted in an enrichment of cells with grossly enlarged nuclei with multiple highly extended neurites (Fig 1A-c). Differentiation on PA6 in the presence of both 0.5μM RA and 1mM dbcAMP resulted in differentiated VSC4.1 cells with both an increased frequency of cells with extended neurites which were significantly longer on day 6 of differentiation (Fig 1A-d). Under these conditions the cells with large nuclei which appeared in the cultures treated with 1mM dbcAMP alone are absent. Based on this we chose to look at a time course of VSC4.1 differentiation on PA6 in the presence of 0.5μM RA and 1mM dbcAMP in more detail. VSC4.1 cells do not express Neurofilament-M in their undifferentiated state and none of the culture conditions tested were able to induce the expression on day six (Fig 1A-e, 1A-f, 1A-g and 1A-h).

**Fig 2:**
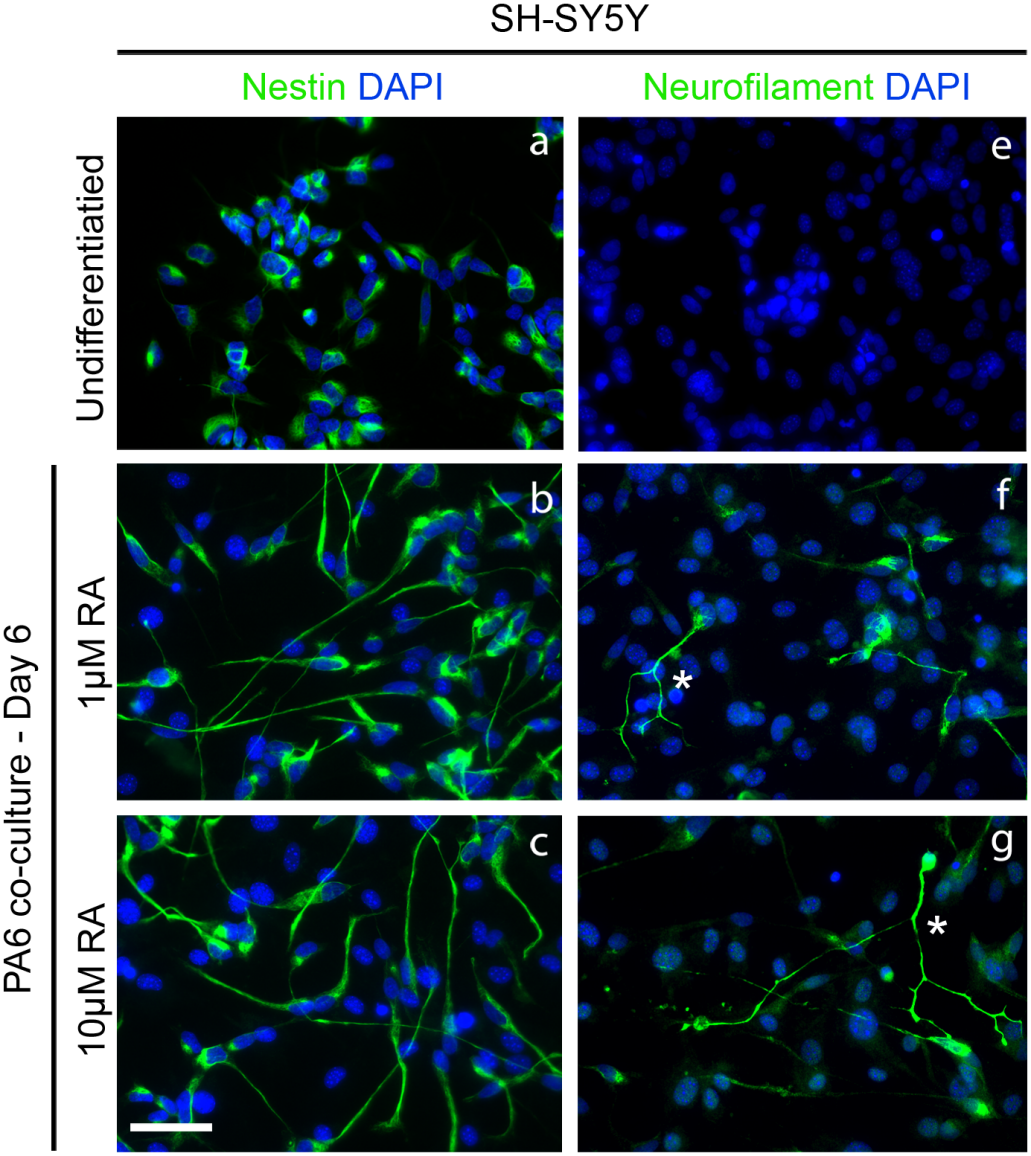
Comparison of differing RA concentrations on SH-SY5Y differentiating on PA6. Undifferentiated SY5Y express nestin but not neurofilament (a, e). Differentiation on PA6 with 1μM or 10μM RA results in the extension of nestin positive processes by day six (b, c). A proportion of these extended neurites are neurofilament positive. More neurites are neurofilament positive at 10μM RA (g) compared to 1μM (f). Scale bar 50μm.

Undifferentiated SH-SY5Y appear to express very low levels of peripherin similar to that seen with VSC4.1. (Fig 1B-a). Expression does not increase even after six days of differentiation on PA6 in the presence of 0.5μM RA (Fig 1B-b), 1mM dbcAMP (Fig 1B-c) or both (Fig 1B-d). As with VSC4.1, SH-SY5Y do not express Neurofilament-M in the undifferentiated state and none of the conditions initially tested induced expression even at day six (Fig 2A-e, 2A-f, 2A-g and 2A-h).

In order to improve the differentiation of SH-SY5Y on PA6 we utilised the same approach as earlier but with higher concentrations of RA (Fig 2) which has been previously shown to induce neurite outgrowth. Undifferentiated SH-SY5Y express nestin (Fig 2-a), an intermediate filament marker of immature neurons. This marker was used to observe neurite extension from day zero onwards while the mature neuronal marker Neurofilament-M (NF-M) was used to identify differentiated SH-SY5Y. By day six of co-culture of SH-SY5Y with PA6 in 1μM and 10μM RA resulted in extensive extension of nestin positive neurites in both cases (Fig 2-b and 2-c). Around 70–80% of cells had neurites substantially longer than those of undifferentiated SH-SY5Y. Again, no NF-M expression was seen in undifferentiated SH-SY5Y (Fig 2-e) but by day six between 40–50% of cells in the RA treated PA6 co-cultures became NF-M positive. In the presence of 1μM RA (Fig 2-f) 10% cells showed strong expression of NF-M in highly extended and branched neurites (Fig 2-f asterisk). This increased to between 50–60% in co-cultures treated with 10μM RA (Fig 2-g). Neurites with strong NF-M expression and substantially longer neurites were seen more frequently in the presence of 10μM RA than at 1μM RA (Fig 2-g asterisk). Thus the optimal condition for differentiation of SH-SY5Y is co-culture on PA6 with 10μM RA.

**Fig 3:**
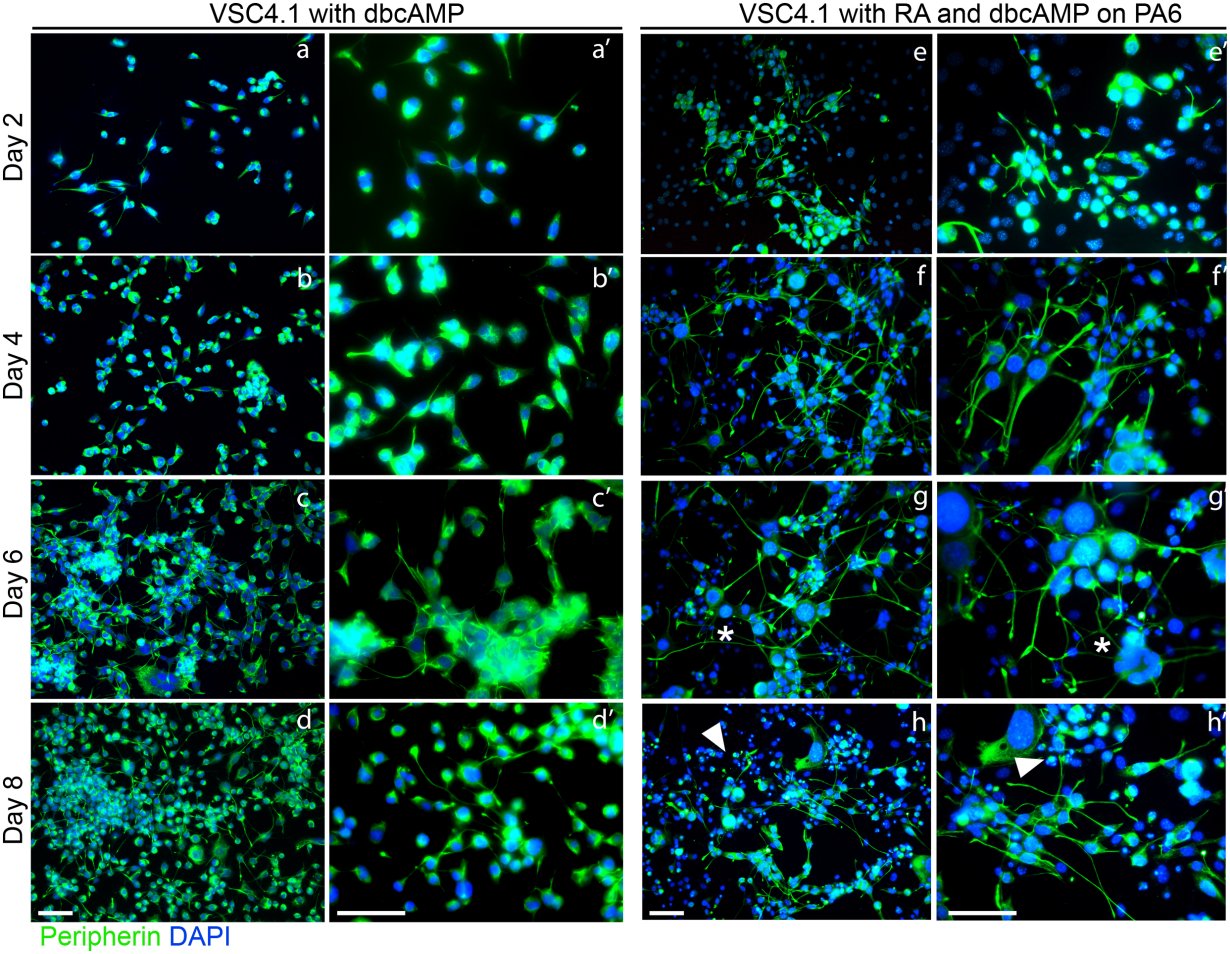
Differentiation of VSC4.1 on PA6 under optimised conditions. Immunostaining for peripherin in a time course of differentiation of VSC4.1 with dbcAMP alone or in combination with RA and PA6. In the absence of RA and PA6 (a-d), short peripherin positive neurites and continued proliferation are seen. Differentiation with RA and dbcAMP on PA6 (e-h) resulted in reduced proliferation and extensive neurite projection. Differentiated VSC4.1 also appear to have slightly enlarged nuclei and thicker neurites as the differentiation progresses. (a’-h’) higher magnifications of the same time point. Scale bars 50μm.

We also compared the differentiation of VSC4.1 with and without PA6 co-culture. Differentiation on PA6 using media containing 0.5μm RA and 1mM dbcAMP resulted in neurites which were longer as compared to those in both undifferentiated and VSC4.1 differentiated by dbcAMP alone by day two (Fig 3-a and 3-e) and most neurites appear to have doubled in length by day four (Fig 3-b and 3-f). Peripherin is seen concentrated in the tips of extending neurites and in a basket like distribution in the cell body. The majority of cells on PA6 on days two and four had one to two neurites with few branches (Fig 3-e and 3-f) while those with dbcAMP alone had a single short major neurite (Fig 3-a and 3-b). On day six neurites extended further and appeared to branch more frequently than at previous time points (Fig 3-c and 3-g asterisks) but substantially more branched neurites were seen in VSC4.1 on PA6. By day eight, some peripherin positive debris and highly condensed nuclei are present indicating some degeneration (Fig 3-h arrows). Neurites that survived on day eight were not as well developed as those seen on day six but remained substantially longer in the PA6 co-culture. During differentiation with dbcAMP alone in the absence of PA6, there was some neurites extensions, however, the VSC4.1 continued to proliferate to a much greater degree than in those cultures differentiated by PA6 co-culture.

**Fig 4:**
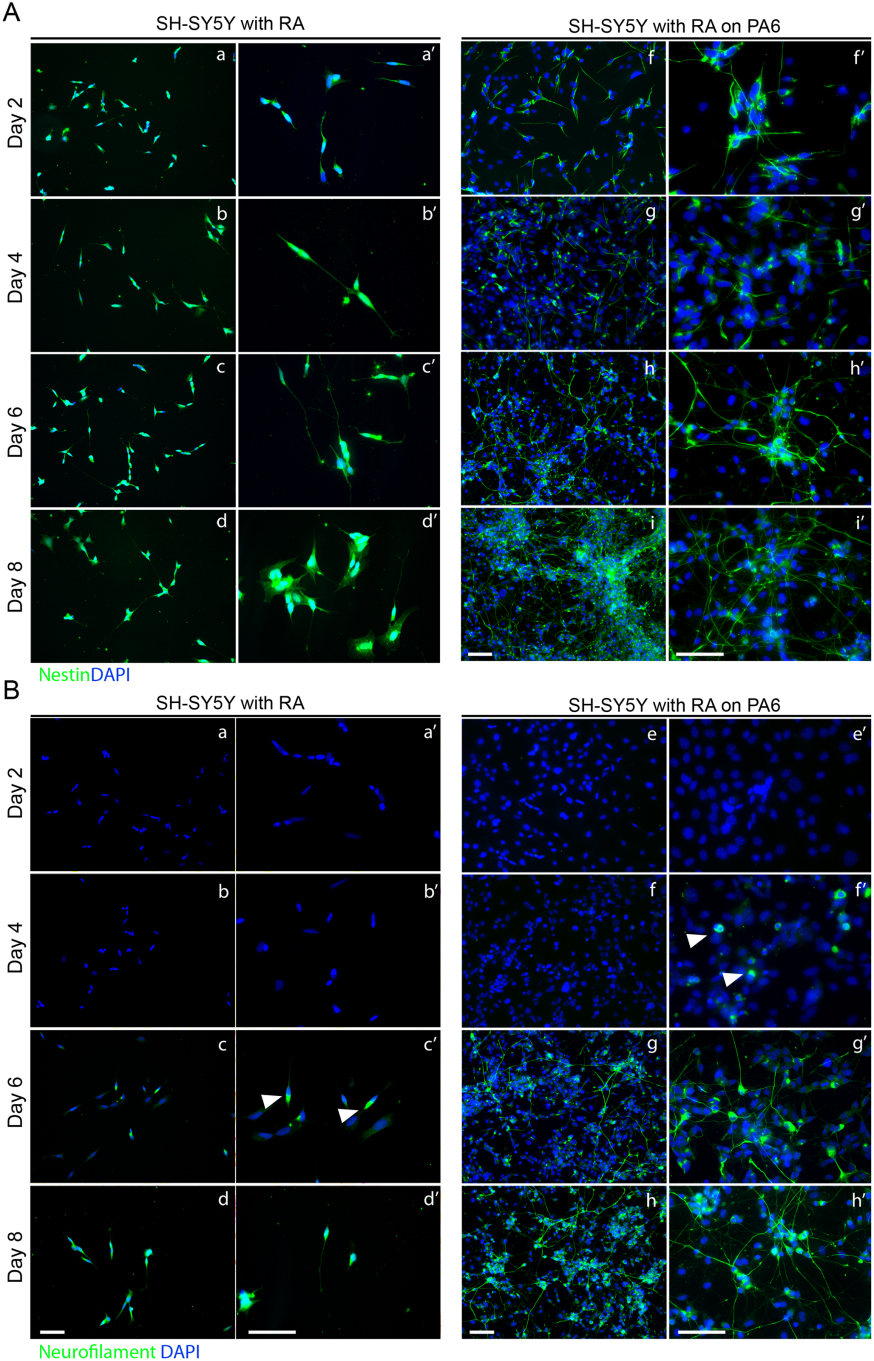
Differentiation of SY5Y on PA6 under optimised conditions. (A) Neurites are already considerably extended by day two when compared to RA alone (f) with much more extensive networks can be seen by day eight (i). Nestin staining appears less intense on day eight compared to day six (h) potentially indicating expected decreased expression on maturation. (B) Neurofilament expression can be seen first in the cell body on day six of differentiation without PA6 (c, arrows). With PA6 however, this is first seen on day four (f, arrows). Neurofilament is found in neurites of increasing length between day six and eight with PA6 (g to h). This also occurs on day eight without PA6 to a lesser degree (d). (a’-h’) higher magnifications of the same time point. Scale bars 50μm.

Differentiation of SH-SY5Y on PA6 in media containing 10μm RA resulted in the extension of neurites on day two, as seen by nestin staining (Fig 4A-f) but in comparison little neurite extension was seen in SH-SY5Y treated with RA alone (Fig 4A-a). Small clusters of non-filamentous nestin were observed in the cell body and within neurites. Nestin continued to be expressed throughout the observed time course without change in levels (Fig 4Aa–4Ad and 4Af–4Ai). Non-filamentous NF-M was present within cell bodies by day four in co-culture with PA6 (Fig 4B-f arrows) but was absent in monoculture until day six (Fig 4B-c arrows). NF-M was subsequently seen as filaments in SH-SY5Y derived neurites by day six or day eight, with and without PA6 respectively (Fig 4B-g and 4B-d). NF-M staining typically appears stronger in longer neurites in both cases. Cells with shorter neurites had more neurofilament in the cell body. By day eight of differentiation on PA6 the number of NF-M positive cells increased and neurites were longer with infrequent branching (Fig 4B-h). As on day six, cells with shorter neurites had more NF-M in the cell body and these were also more frequent. By day eight of differentiation without PA6 there was considerable cell death but those cells remaining possess NF-M positive neurite extensions substantially short than those seen with PA6 (Fig 4B-d).

**Fig 5:**
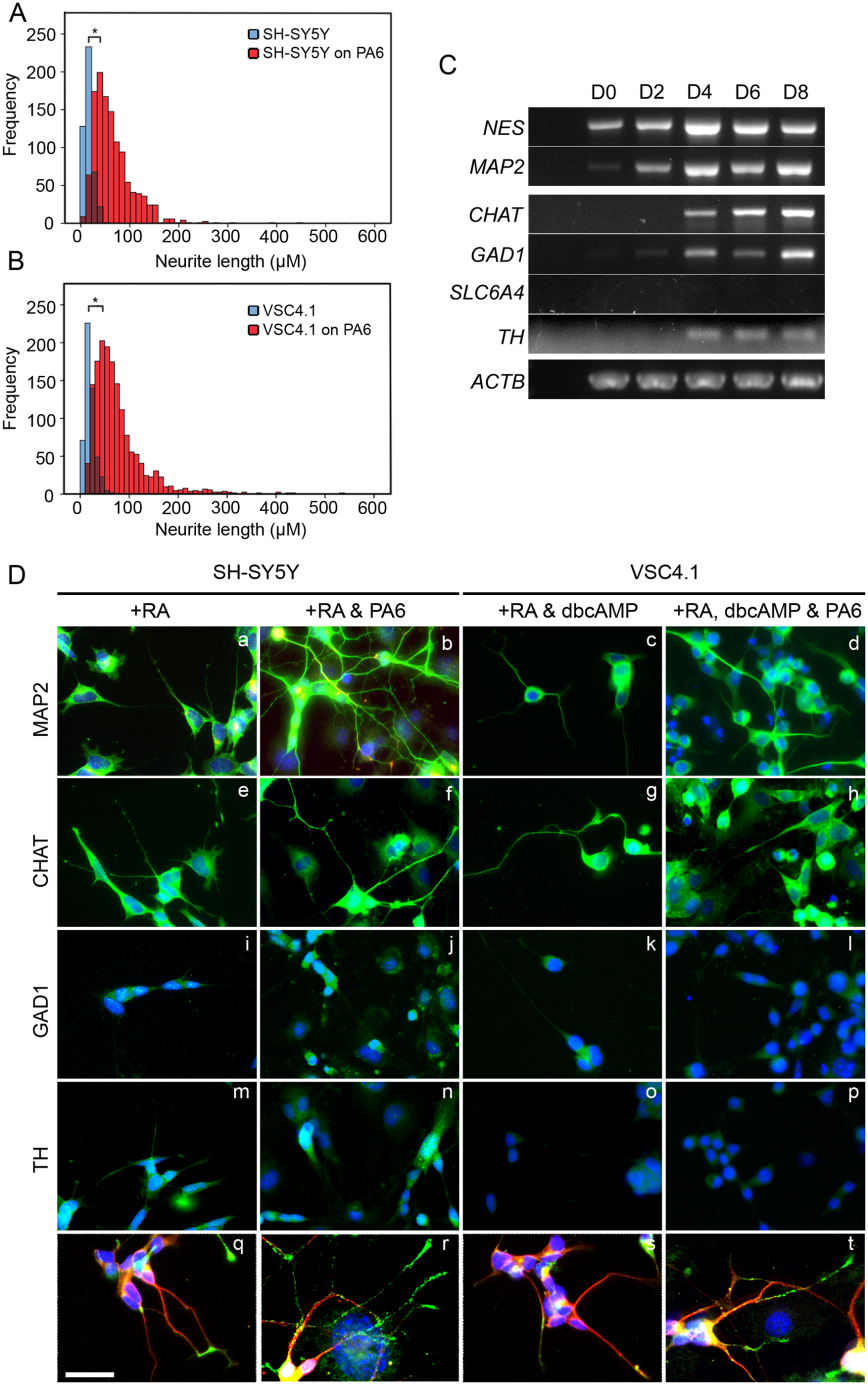
Characterisation of SH-SY5Y and VSC4.1 differentiated on PA6 under optimised conditions. (A) Differentiation of VSC4.1 on PA6 with 0.5μM RA and 1mM dbcAMP (VSC4.1 on PA6) results in neurites which are significantly longer and more numerous than VSC4.1 differentiated with 1mM dbcAMP alone (VSC4.1 alone). (B) Similarly, differentiation of SY5Y in the presence of PA6 and 10uM RA (SY5Y on PA6) results in neurites which are significantly longer and more numerous than those present when differentiated with 10uM RA alone (SY5Y alone). (A and B) Comparison of neurite lengths on day six between differentiation protocols with and without PA6. Student’s t-tests were performed on log transformed data. * P<0.05. VSC4.1 alone n = 517, VSC4.1 on PA6 n = 1581, SY5Y alone n = 345, SY5Y on PA6 n = 740. (C) RT-PCR for transcripts associated with immature (Nestin), mature (MAP2), cholinergic (CHAT), GABAergic (GAD1), serotonergic (SLC6A4) and dopaminergic (TH) neurons using ACTB as a loading control over eight days of differentiation on PA6. (-) Negative control. (D) Immunostaining to confirm the expression of markers identified by RT-PCR in SH-SY5Y or VSC4.1 differentiated with or without PA6 for eight days. Scale bar 50μm.

We measured and quantified neurite length was measured on day six of VSC4.1 differentiation and day eight of SH-SY5Y differentiation both in mono and co-culture with PA6. SH-SY5Y differentiated on PA6 with RA had significantly longer neurites than those differentiated with RA alone with a median length of 64μm or 16μm respectively (Fig 5A). Longer neurites occurred more frequently when differentiation was induced by co-culture on PA6 with 66% being over 50μm while less than 1% of neurites extended beyond this by differentiation with RA alone. Maximum neurite lengths achieved by differentiation in co-culture with PA6 were considerably longer with rare neurites reaching up to 510μm whereas a few neurites in SH-SY5Y differentiated without PA6 reached a maximum of 52μm.We also observed similar results for VSC4.1 Differentiation of VSC4.1 on PA6 with RA and dbcAMP resulted in significantly longer neurites in comparison to neurites generated by dbcAMP alone (Fig 5B). The median length of neurites of VSC4.1 differentiated on PA6 was 61μm compared with 20μm without PA6. As with SH-SY5Y, long neurites were also more frequent with 63% being over 50μm compared to only 2% of neurites after differentiation with dbcAMP alone. Again, maximum lengths of rare neurites reached 538μm with PA6 in contrast to 78μm without.

Expression of Nestin increased from day zero to eight as expected over the course of the differentiation concomitant with increased neurite length when SH-SY5Y cells were differentiated on PA6 with RA (Fig 5C). MAP2 expression was seen from day two onwards, indicating that SH-SY5Y had acquired a more mature identity as they differentiated. RT-PCR for neuronal subtype markers showed increased levels of transcripts associated with three neuronal subtype markers; Cholinergic (*CHAT*), GABAergic (*GAD1)*, Serotonergic (*SLC6A4)* and dopaminergic (*TH*)(Fig 5C). Both *CHAT* and *GAD1* appeared first at low levels on day two and increased up to day eight, *CHAT* levels appear to be higher than *GAD1* from day six to eight. *TH* expression was also seen from day four but at much lower levels and with little change of the remaining time course. No *SLC6A4* expression was seen at any time point.

These results obtained by RT PCR for neuronal subtypes were confirmed by immunostaining of SH-SY5Y on day eight of differentiation in the presence of RA both with and without PA6 co-culture (Fig 5D). As expected MAP2 was present under both conditions however it appeared much more extensive after differentiation with PA6 and in keeping with the increased neurite lengths (Fig 5Da and 5Db). The majority of differentiated SH-SY5Y were ChAT positive under both conditions but again levels appear to be increased after differentiation with PA6 (Fig 5De and 5Df). GAD1 and TH positive SH-SY5Y were present also under both conditions but at much lower frequencies than CHAT positive cells (Fig 5Di, 5Dj, 5Dm and 5Dn).

The VSC4.1 differentiated neurons expressed MAP2 in both with and without PA6 but again a more extensive network was observed in the PA6 co-cultures (Fig 5Dc and 5Dd). As with SH-SY5Y, the majority of differentiated VSC4.1 cells were CHAT positive (Fig 5Dg and 5Dh) but were negative for GAD1 or TH (Fig 5Dk, 5Dl, 5Do and 5Dp). Both SH-SY5Y and VSC4.1 cells expressed the synaptic vesicle marker SV2 under both differentiation conditions. However, compared to cells differentiated in the small molecules alone, cells differentiated with small molecules and in PA6 co-cultures, the expression of SV2 was stronger and with a higher proportion of cells staining positive (Fig 5Dq–5Dt). Additionally in cells differentiated on PA6, the SV2 positive vesicles appeared strongest in neurites with reduced MAP2 levels indicating the presence of a mature axon.

**Fig 6:**
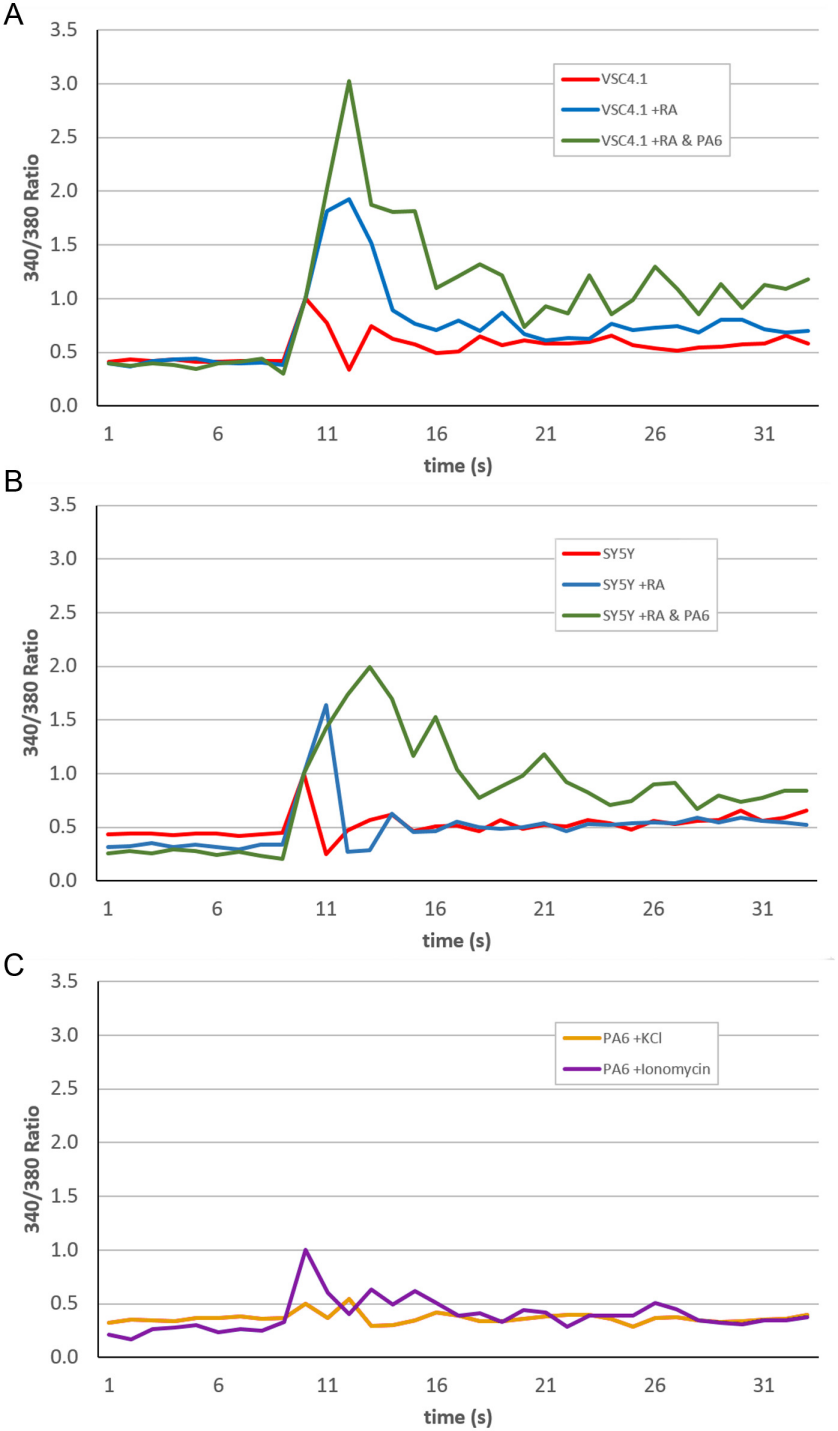
PA6 co-culture results in increased calcium influx in response to stimulus. Ratio of Fura-2 fluorescence emission after 340nm and 380nm excitation in (A) VSC4.1 (B) SY5Y; In both cases cell lines assayed include undifferentiated or differentiated with or without PA6, (C) PA6 alone. Wells were assayed over 40s with stimulus addition at 10s. Stimulus was 50mM KCl and 2μM ionomycin was used as a positive control. Ratios calculated from mean of triplicate wells, representative data from two independent experiments.

The ability of the differentiated SH-SY5Y neurons to respond to stimuli by firing action potentials was determined by the observation of Fura-2 loaded cells. Stimulation of undifferentiated VSC4.1 with 50mM KCl resulted in a transient small increase in intracellular calcium levels (Fig 6A and 6B). This fold increase rises to nearly four-fold and three-fold respectively in VSC4.1 and SY5Y differentiated without PA6 while co-culture results in a further six-fold and four-fold peak in intracellular calcium levels respectively. Additionally, calcium flux appears prolonged where differentiation occurs in the presence of PA6 with increased intracellular calcium levels seen following stimulation when compared to undifferentiated cells and those differentiated alone. The PA6 cells alone do not respond to stimulation by 50mM KCl and therefore do not contribute to the flux seen in the co-culture system, however changes in intracellular calcium levels can be induced with the addition of 2μM ionomycin (Fig 6C).

**Fig 7:**
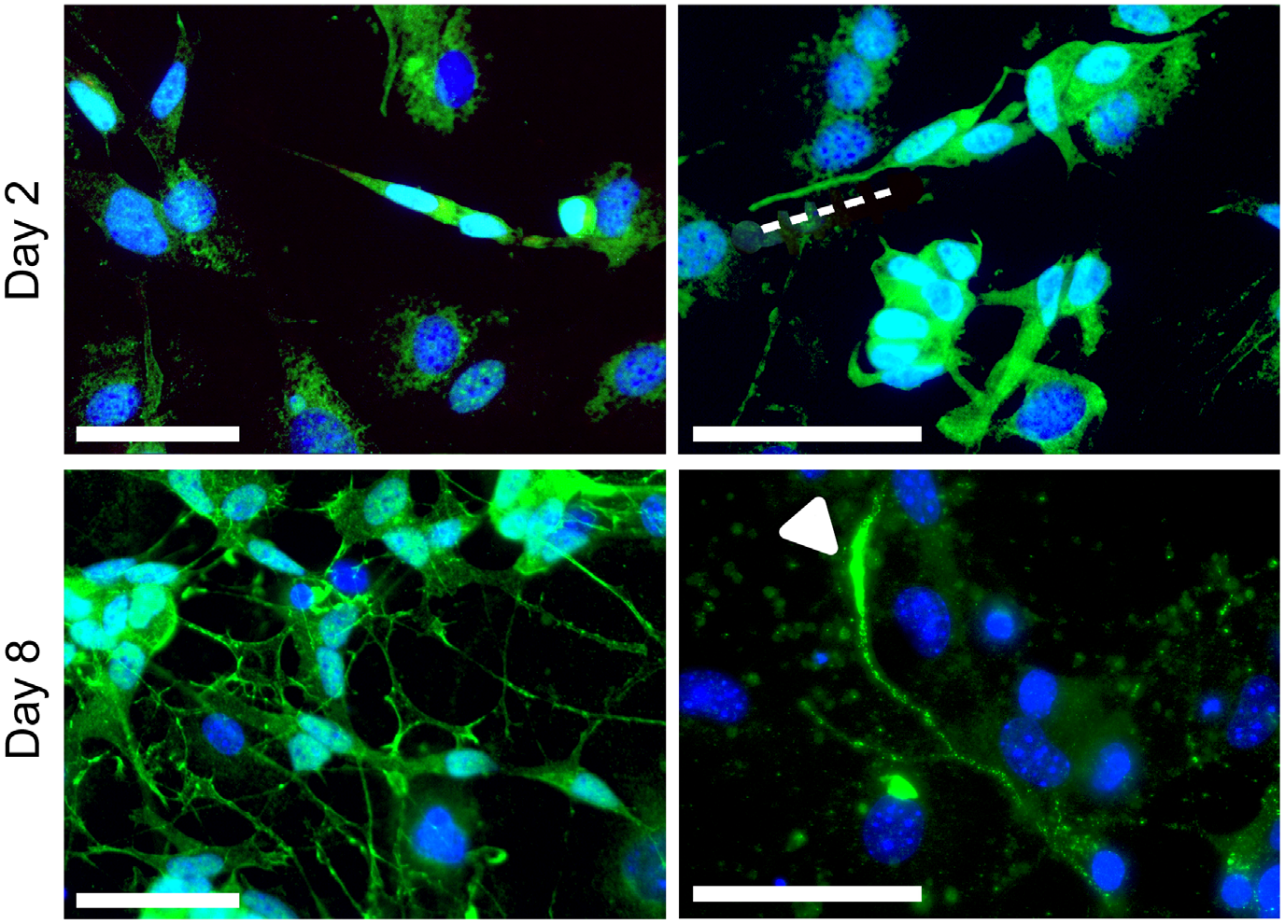
Differentiation of SH-SY5Ycells expressing HA tagged mouse angiogenin on PA6. (a-b) SH-SY5Y on PA6 on Day 2 continue to show uniform expression in cytoplasm and neurite. (c-d) By day eight expression is reduced at the base of the neurite, but greatly increased at the tip.Scale bar (50μm).

Having established optimal differentiation conditions to generate to mature neurons for both SH-SY5Y and VSC4.1 cell lines we studied the expression and distribution of a neurodegenerative disease associated protein angiogenin. For this we used a stable SH-SY5Y cell line which constitutively expresses a HA-tagged mouse angiogenin 1 (Ang-HA) which we had generated previously (23). Ang-HA is found uniformly throughout the cytoplasm of the cell body and short neurite of undifferentiated SH-SY5Y in small granular vesicle-like structures (Fig 7a and 7b). Upon differentiation its distribution in the cytoplasm surrounding the nucleus and the proximal part of the extending neurite was retained. However, levels within the neurite its self were substantially increased and a large pool of mAng1HA was seen in the tips of extended neurites (Fig 7c and 7d) in keeping with its predicted role in neurite extension [25]

## Discussion

Differentiation of SH-SY5Y or VSC4.1 on PA6 offers an *in vitro* model system suitable for the study of many aspects of neurological development and disease though with critical limitations. We have shown co-culture with PA6 in combination with external factors (RA with SH-SY5Y and RA with dbcAMP with VSC4.1) results in the acquisition of mature neuronal characteristics such as increased neurofilament expression and neurite length, with both increased frequency and neurite length at earlier time points, when compared to mono-culture differentiation. This is not to say that the SH-SY5Y cell line is the best model for studying neurodegenerative disease. However, as it is a well-established cell line in the field and still used very extensively, the co-culture differentiation method we report herein, resulting in a more mature phenotype is a valuable tool. Our argument for the use of this model also stems from logistics, as it is considerably faster, cheaper and requires less technical skill to culture than primary or ES-derived neurons. These characteristics make it a strong candidate for exploratory cell biology studies.

The core components of the PA6 SDIA have been defined as stromal cell-derived factor 1 (SDF-1), pleiotrophin (PTN), insulin-like growth factor (IGF2 and ephrin B1 (EFNB1), collectively termed SPIE factors by Vazin [26]. In addition to this multiple groups have identified Wnt5a, sonic hedgehog (Shh), secreted frizzled-related protein 1 (sFRP1) and vascular endothelial growth factor D (VEGFD) as contributing factors to SDIA [27]. Many modified SH-SY5Y differentiation protocols include additional neural-inducing cytokines. Encinas et al., [15] sequentially treated SH-SY5Y with RA for five days followed by brain-derived neurotrophic factor (BDNF) and reported withdrawal of cells from the cell cycle in addition to mature neuronal markers such as NF, neuron-specific enolase and growth associated protein 43. It was also shown that RA-BDNF treated cells exhibit carbachol-evoked noradrenaline release but no significant choline acetyltransferase activity. While there have been no reports of BDNF secretion from PA6 directly, there are reports of its secretion in combination with nerve growth factor (NGF) from adipose-derived stem cells at concentrations capable of directing neural differentiation [28]. Therefore other factors specific to PA6 may be directing SH-SY5Y to differentiate to a mostly cholinergic identity in combination with RA. Differentiation with RA in conjunction with other factors (such as TPA) or also appears to drive SH-SY5Y towards dopaminergic/noradrenergic while RA alone leads to cholinergic identities [7,12,13]. Confusingly, recent transcriptional analysis of SH-SY5Y differentiated with RA alone has shown both increased expression of dopaminergic markers and suppression of serotonergic, cholinergic and noradrenergic characteristics [29]. Though this was after only eight days of differentiation with 1μM (unlike the 10μM RA conventionally used) it demonstrates care should be taken to properly identify the cell types present.

SH-SY5Y cultures typically contain both neuroblast-like cell morphologies (N-type) and rarer epithelial-like (E-type), transdifferentiation can occur between the two [30]. During differentiation without PA6 more E-type cells remain but do not appear to increase in number, presumably due the lack of serum. During differentiation on PA6 however, these E-type cells appear to be absent. Very few E-type cells are present in our undifferentiated cultures initially therefore it may be that N-type SH-SY5Y transdifferentiate to E-type upon seeding without PA6 or vice-versa and the reduced cell numbers due to the death of N-type cells at later time points without PA6 leaves the initial E-type cells behind.

Little work has been done to characterise VSC4.1 both before and after differentiation with dbcAMP alone so little speculation can be made as to the effects of RA and PA6 beyond the observation that neurites extend further and more frequently and fewer enlarged nuclei are present in addition to elevated CHAT levels within differentiated cells. As RA has been shown to direct exit from the cell cycle in other neuroblastoma it may indicate the grossly enlarged nuclei observed with PA6 and dbcAMP are due to cells being unable to divide and becoming multinucleated. The combination of RA and dbcAMP on PA6 clearly inhibits cell division when compared to dbcAMP alone without PA6. It is curious as to why VSC4.1 with dbcAMP alone remain capable of normal (but slowed) division while the addition of PA6 causes these issues but it seems likely this is due to VSC4.1 being a hybridoma rather than a true neuroblastoma and may be responding to secreted cytokines.

Differentiated SH-SY5Y expressing the neurodegenerative disease-associated protein mouse angiogenin 1 showed a striking difference in the distribution of the protein when compared to undifferentiated SH-SY5Y. The concentration in the tip of the neurite after differentiation recapitulates the distribution seen in differentiating P19 EC cells as previously described [22], further highlighting the importance of differentiation to a mature phenotype and the potential for the system to be used as model in studying neurodegenerative disease.

Co-culture methods with PA6 are therefore capable of delivering the required cocktail of neural-inducing factors in a simple, single step manner requiring no concern for the purchase or storage of cytokines, for the preparation of complex differentiation mediums or multiple media changes. In addition to this differentiation appears accelerated, reducing the time required to obtain cells sufficiently differentiated for experimental use. While PA6 has been shown to work efficiently with both mouse and human embryonic stem cells and carcinoma cell lines these still required more culture time and are substantially more complicated to maintain prior to differentiation when compared to either SH-SY5Y or VSC4.1. Additionally as neuroblastoma are proliferative and clonal a nearly unlimited number of cells can be obtained unlike other sources of neurons such as primary cultures which are limited in number and a mixed population of neurons and other cell types. These co-culture methods can easily and rapidly provide cells with identities very close to those of mature primary neurons.

## References

[1] Cheung Y-T, Lau WK-W, Yu M-S, Lai CS-W, Yeung S-C, So K-F, et al Effects of all-trans-retinoic acid on human SH-SY5Y neuroblastoma as in vitro model in neurotoxicity research. Neurotoxicology. 2009;30: 127–135. 10.1016/j.neuro.2008.11.001 19056420

[2] Storch A, Burkhardt K, Ludolph AC, Schwarz J. Protective effects of riluzole on dopamine neurons: involvement of oxidative stress and cellular energy metabolism. J Neurochem. 2000;75: 2259–2269. 11080177 10.1046/j.1471-4159.2000.0752259.x

[3] Knaryan VH, Samantaray S, Park S, Azuma M, Inoue J, Banik NL. SNJ-1945, a calpain inhibitor, protects SH-SY5Y cells against MPP ^+^ and rotenone. J Neurochem. 2013; n/a–n/a. 10.1111/jnc.12629 PMC4038676 24341912

[4] Biedler JL, Helson L, Spengler BA. Morphology and Growth, Tumorigenicity, and Cytogenetics of Human Neuroblastoma Cells in Continuous Culture. Cancer Res. 1973;33: 2643–2652. 4748425

[5] Ciccarone V, Spengler BA, Meyers MB, Biedler JL, Ross RA. Phenotypic Diversification in Human Neuroblastoma Cells: Expression of Distinct Neural Crest Lineages. Cancer Res. 1989;49: 219–225. 2535691

[6] Inoka S, Wimalasena K. Are SH-SY5Y and MN9D cell lines truly dopaminergic? FASEB J. 2007;21: 912–919.

[7] Xie H, Hu L, Li G. SH-SY5Y human neuroblastoma cell line: in vitro cell model of dopaminergic neurons in Parkinson’s disease. Chin Med J (Engl). 2010;123: 1086–1092. 20497720

[8] Alexianu ME, Mohamed AH, Smith RG, Colom LV, Appel SH. Apoptotic Cell Death of a Hybrid Motoneuron Cell Line Induced by Immunoglobulins from Patients with Amyotrophic Lateral Sclerosis. J Neurochem. 1994;63: 2365–2368. 10.1046/j.1471-4159.1994.63062365.x 7964760

[9] Smith RG, Alexianu ME, Crawford G, Nyormoi O, Stefani E, Appel SH. Cytotoxicity of immunoglobulins from amyotrophic lateral sclerosis patients on a hybrid motoneuron cell line. Proc Natl Acad Sci U S A. 1994;91: 3393–3397. 8159758 10.1073/pnas.91.8.3393 PMC43583

[10] Påhlman S, Ruusala AI, Abrahamsson L, Odelstad L, Nilsson K. Kinetics and concentration effects of TPA-induced differentiation of cultured human neuroblastoma cells. Cell Differ. 1983;12: 165–170. 6299586 10.1016/0045-6039(83)90006-4

[11] Adem A, Mattsson ME, Nordberg A, Påhlman S. Muscarinic receptors in human SH-SY5Y neuroblastoma cell line: regulation by phorbol ester and retinoic acid-induced differentiation. Brain Res. 1987;430: 235–242. 3607514 10.1016/0165-3806(87)90156-8

[12] Presgraves SP, Ahmed T, Borwege S, Joyce JN. Terminally differentiated SH-SY5Y cells provide a model system for studying neuroprotective effects of dopamine agonists. Neurotox Res. 2004;5: 579–598. 15111235 10.1007/BF03033178

[13] Påhlman S, Ruusala AI, Abrahamsson L, Mattsson ME, Esscher T. Retinoic acid-induced differentiation of cultured human neuroblastoma cells: a comparison with phorbolester-induced differentiation. Cell Differ. 1984;14: 135–144. 6467378 10.1016/0045-6039(84)90038-1

[14] Constantinescu R, Constantinescu AT, Reichmann H, Janetzky B. Neuronal differentiation and long-term culture of the human neuroblastoma line SH-SY5Y. J Neural Transm Suppl. 2007; 17–28. 17982873 10.1007/978-3-211-73574-9_3

[15] Encinas M, Iglesias M, Liu Y, Wang H, Muhaisen A, Ceña V, et al Sequential Treatment of SH-SY5Y Cells with Retinoic Acid and Brain-Derived Neurotrophic Factor Gives Rise to Fully Differentiated, Neurotrophic Factor-Dependent, Human Neuron-Like Cells. J Neurochem. 2000;75: 991–1003. 10.1046/j.1471-4159.2000.0750991.x 10936180

[16] Lyuh E, Kim H-J, Kim M, Lee J-K, Park K-S, Yoo K-Y, et al Dose-specific or dose-dependent effect of growth hormone treatment on the proliferation and differentiation of cultured neuronal cells. Growth Horm IGF Res. 2007;17: 315–322. 10.1016/j.ghir.2007.03.002 17482859

[17] Kodama H-A, Amagai Y, Koyama H, Kasai S. Hormonal responsiveness of a preadipose cell line derived from newborn mouse calvaria. J Cell Physiol. 1982;112: 83–88. 10.1002/jcp.1041120113 7107717

[18] Kawasaki H, Mizuseki K, Nishikawa S, Kaneko S, Kuwana Y, Nakanishi S, et al Induction of Midbrain Dopaminergic Neurons from ES Cells by Stromal Cell–Derived Inducing Activity. Neuron. 2000;28: 31–40. 10.1016/S0896-6273(00)00083-0 11086981

[19] Zeng X, Cai J, Chen J, Luo Y, You Z-B, Fotter E, et al Dopaminergic differentiation of human embryonic stem cells. Stem Cells Dayt Ohio. 2004;22: 925–940. 10.1634/stemcells.22-6-925 15536184

[20] Lee SH, Lumelsky N, Studer L, Auerbach JM, McKay RD. Efficient generation of midbrain and hindbrain neurons from mouse embryonic stem cells. Nat Biotechnol. 2000;18: 675–679. 10.1038/76536 10835609

[21] Kwon Y-W, Chung Y-J, Kim J, Lee H-J, Park J, Roh T-Y, et al Comparative study of efficacy of dopaminergic neuron differentiation between embryonic stem cell and protein-based induced pluripotent stem cell. PloS One. 2014;9: e85736 10.1371/journal.pone.0085736 24465672 PMC3899054

[22] Subramanian V, Crabtree B, Acharya KR. Human angiogenin is a neuroprotective factor and amyotrophic lateral sclerosis associated angiogenin variants affect neurite extension/pathfinding and survival of motor neurons. Hum Mol Genet. 2008;17: 130–149. 10.1093/hmg/ddm290 17916583

[23] Thiyagarajan N, Ferguson R, Subramanian V, Acharya KR. Structural and molecular insights into the mechanism of action of human angiogenin-ALS variants in neurons. Nat Commun. 2012;3: 1121 10.1038/ncomms2126 23047679 PMC3493651

[24] Greenway MJ, Alexander MD, Ennis S, Traynor BJ, Corr B, Frost E, et al A novel candidate region for ALS on chromosome 14q11.2. Neurology. 2004;63: 1936–1938. 15557516 10.1212/01.wnl.0000144344.39103.f6

[25] Subramanian V, Feng Y. A new role for angiogenin in neurite growth and pathfinding: implications for amyotrophic lateral sclerosis. Hum Mol Genet. 2007;16: 1445–1453. 10.1093/hmg/ddm095 17468498

[26] Vazin T, Becker KG, Chen J, Spivak CE, Lupica CR, Zhang Y, et al A Novel Combination of Factors, Termed SPIE, which Promotes Dopaminergic Neuron Differentiation from Human Embryonic Stem Cells. PLoS ONE. 2009;4: e6606 10.1371/journal.pone.0006606 19672298 PMC2719871

[27] Guloglu M o., Larsen A, Brundin P. Adipocytes derived from PA6 cells reliably promote the differentiation of dopaminergic neurons from human embryonic stem cells. J Neurosci Res. 2014;92: 564–573. 10.1002/jnr.23355 24482287

[28] Razavi S, Razavi MR, Kheirollahi-Kouhestani M, Mardani M, Mostafavi FS. Co-culture with neurotrophic factor secreting cells induced from adipose-derived stem cells: promotes neurogenic differentiation. Biochem Biophys Res Commun. 2013;440: 381–387. 10.1016/j.bbrc.2013.09.069 24064351

[29] Korecka JA, van Kesteren RE, Blaas E, Spitzer SO, Kamstra JH, Smit AB, et al Phenotypic Characterization of Retinoic Acid Differentiated SH-SY5Y Cells by Transcriptional Profiling. PLoS ONE. 2013;8: e63862 10.1371/journal.pone.0063862 23724009 PMC3665836

[30] Ross RA, Spengler BA, Biedler JL. Coordinate morphological and biochemical interconversion of human neuroblastoma cells. J Natl Cancer Inst. 1983;71: 741–747. 6137586

